# Hernioscopy: A Description of an Adjunct Technique in Emergency Femoral Hernia Surgery

**DOI:** 10.7759/cureus.35314

**Published:** 2023-02-22

**Authors:** Paul W Shuttleworth, Shariq Sabri, Ehtisham Zeb, Andrei Mihailescu

**Affiliations:** 1 General Surgery, Tameside General Hospital, Manchester, GBR

**Keywords:** diagnostic laparoscopy, femoral hernia, general emergency surgery, groin hernia, minimally invasive surgery

## Abstract

Femoral hernias commonly present as an emergency with a large proportion strangulated or with contents that are threatened. Many surgical options are available including minimally invasive surgery and multiple open approaches. A low approach allows a relatively simple repair of the hernia and has a long-established history of safety with reproducible outcomes and low recurrence rates. It is technically less challenging than a high approach but does not allow easy assessment or management of hernia sac contents. We highlight and describe a technique that can be used when the hernia reduces spontaneously at induction, or when the surgeon cannot be confident that the contents are viable. Hernioscopy is the technique of utilizing a laparoscope inserted via the hernia sac to either examine the abdominal contents or facilitate the safe creation of pneumoperitoneum and further insertion of ports transabdominally when the patient has pelvic adhesions. We describe the operative steps taken to make this a feasible approach and reduce the need for unnecessary laparotomies and the associated morbidity.

## Introduction

Asymptomatic groin hernias are commonly managed via *watchful waiting* [1], with a relatively low risk of strangulation, particularly for inguinal hernias. Factors affecting the risk for strangulation include femoral hernias, female patients, symptomatic hernias, length of symptoms duration, and age [2]. Femoral hernias have a much higher strangulation rate data, and according to the Swedish and Danish hernia registries [3,4], this can be as high as 45%. Incarcerated or complicated hernias are organ and life-threatening conditions that commonly present as an emergency. A significant proportion of these presentations are for incarcerated femoral hernias, with a large proportion presenting for the first time as an emergency.

Hernias that are suspected of containing strangulated contents are operated on as an emergency to release or repair the strangulated contents and repair the hernial defect. It is important to adequately assess the viability of hernial contents at the time of repair. Viable contents can be simply reduced back to the abdominal cavity as opposed to frankly ischemic intestine which requires resection and definitive management.

Several techniques are used contemporaneously to repair femoral hernias, including open or minimally invasive approaches. The majority of the cases are still dealt with via an open (anterior) approach. When open repair is used, three different types of procedures performed via different incisions can be used to approach the femoral canal and hernia sac. These include the high, pre-peritoneal approach (modified McEvedy), trans-inguinal approach (Lotheissen), or low, infra-inguinal approach (Lockwood) [5]. There are advantages and disadvantages to each technique. For the high, McEvedy approach, the advantages include good exposure to the pre-peritoneal structures, including the hernia sac, and the ability to inspect the intra-abdominal viscera, if required. Nevertheless, this technique is associated with higher risks of injury to other organs, including the bladder and epigastric vessels. In comparison, the trans-inguinal and infra-inguinal approaches are technically easier as the Lotheissen approach is similar to a standard inguinal hernia approach, and the Lockwood infra-inguinal procedure approaches the hernia sac directly, with an incision over the femoral canal. However, the contents of the hernia sac are not easily accessible via these approaches as access to the peritoneal cavity is limited by the hernia defect itself and the relatively rigid borders of the femoral ring. In some circumstances, following administration of a general anesthetic, the hernia contents can spontaneously reduce, preventing assessment of its contents via a low approach, and mandating either laparoscopy or laparotomy if a low approach is used.

We highlight a technique that can be utilized to prevent unnecessary laparotomies in cases where the sac contents require direct inspection during a complicated femoral hernia repair, and an infra or trans-inguinal approach has been used. The benefits of minimally invasive surgery are well established with less postoperative pain, earlier return to function, and earlier discharge. The authors propose that this technique can be especially useful in frail, comorbid, and unwell patients with high National Emergency Laparotomy Audit (NELA) and Portsmouth Physiological and Operative Severity Score for the enumeration of mortality and Morbidity (P-POSSUM) scores avoiding unnecessary laparotomies and their associated risk. It is also useful when patients have had multiple previous operations and there is a high suspicion of adhesions.

Hernioscopy is a technique where a laparoscope is inserted directly through the hernia defect using the hernia sac edges to keep the trocar in place. This allows the safe creation of pneumoperitoneum, facilitating inspection of the reduced hernia sac contents. Insertion of transabdominal trocars, if required, can be safely achieved under vision, particularly in patients who have had multiple previous laparotomies and may have significant adhesions. Procedural steps as performed in our unit are presented in this article.

Groin hernia surgery is one of the most commonly performed surgical operations, but femoral hernias are relatively rare. Therefore, it is advisable for all surgeons in training to be aware of this alternative approach in select cases.

## Technical report

Procedural steps

**Figure 1:**
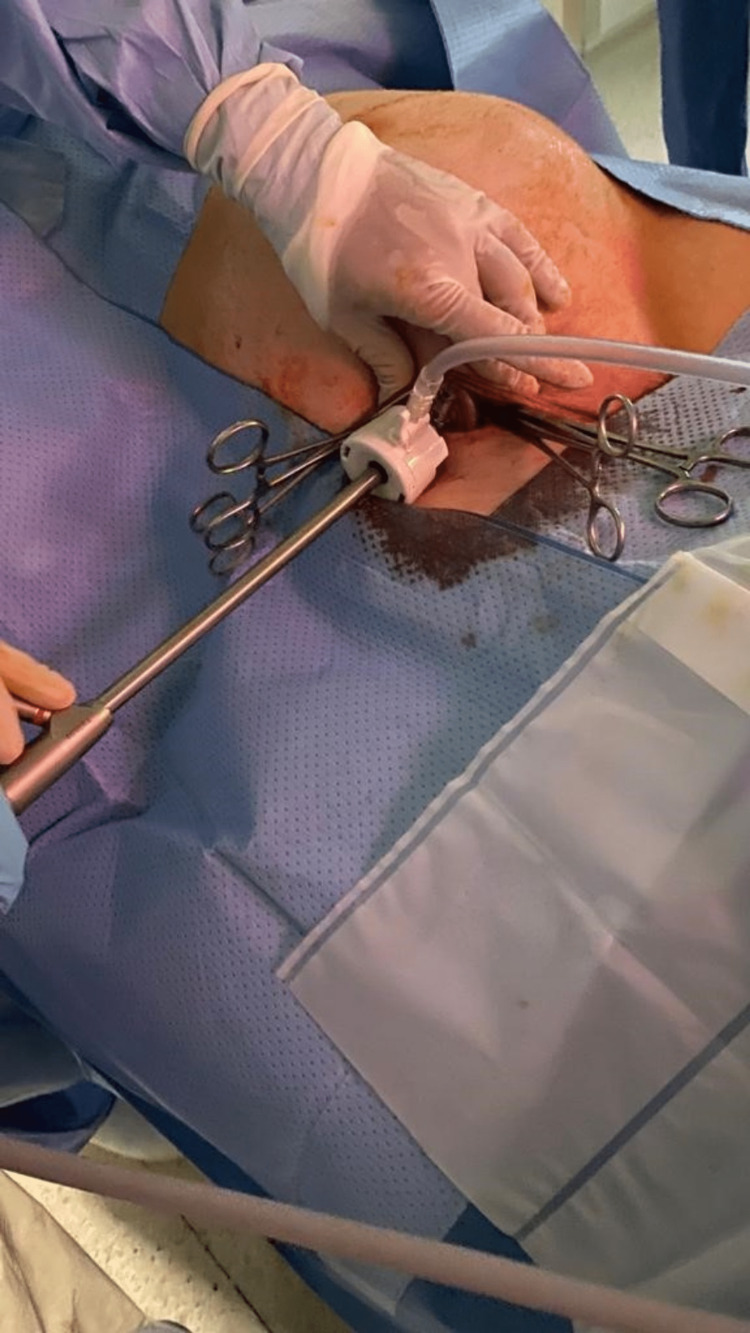
Safe creation of pneumoperitoneum. The figure shows the creation of a safe pneumoperitoneum into which a 10 mm laparoscope is inserted. It also shows the draping we use to give adequate space for abdominal ports to be inserted should they be required.

**Figure 2:**
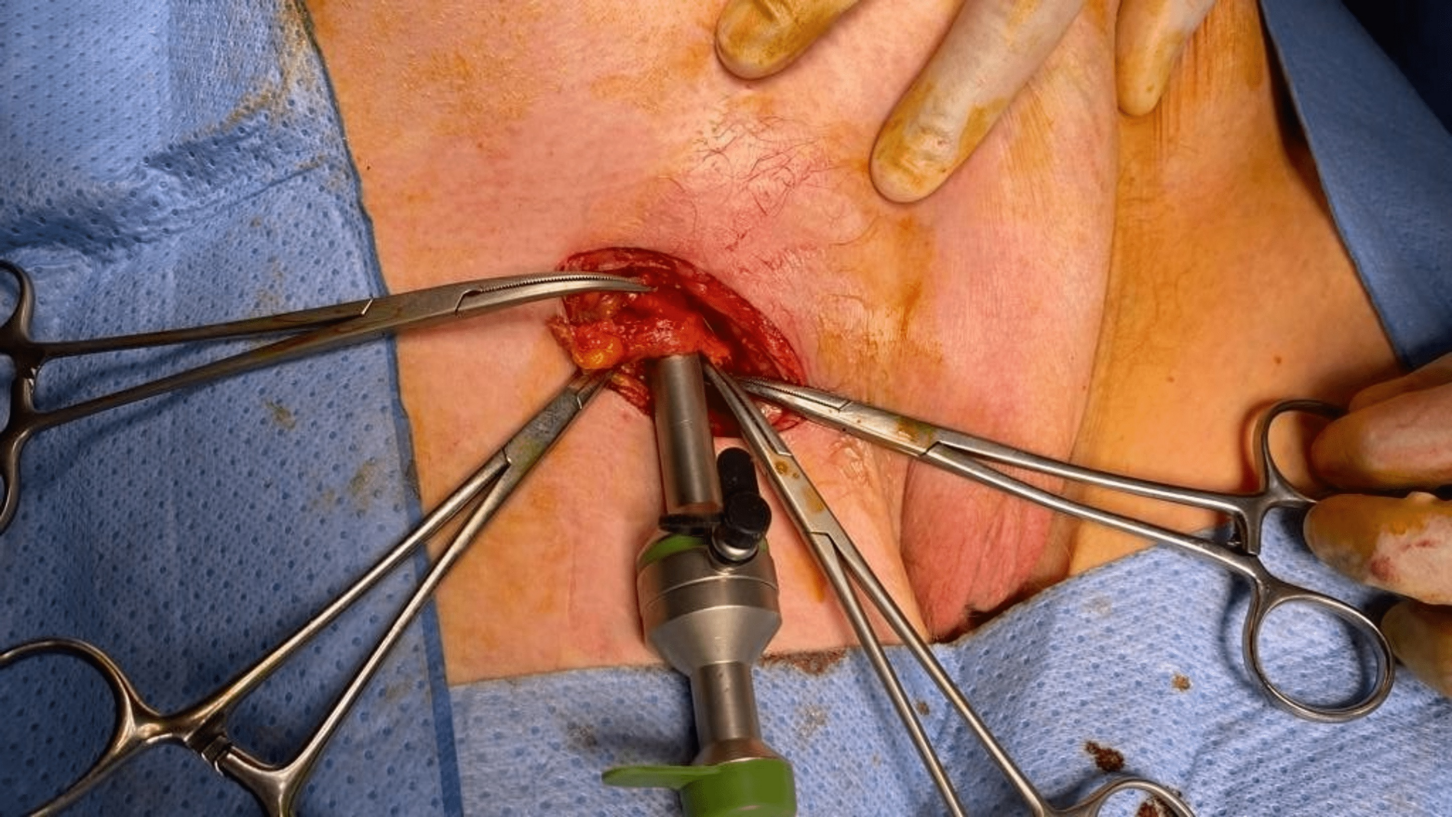
Insertion of a metal trocar through a right femoral hernia defect. Th figure shows the hernia defect controlled between clips with a standard reusable metal trocar inserted.

Using the standard low approach, an incision is made over the hernia sac, as described by Lockwood. The hernia sac is defined and dissected from the surrounding structures using a mixture of sharp dissection and diathermy. Occasionally, there will be incomplete reduction and widening of the femoral ring to fully reduce the hernial contents. If the contents may be retrieved and examined through the defect due to adhesions between the contents and the sac, it is not necessary to proceed with hernioscopy. At the tip of the sac, the peritoneum is checked to ensure the contents are free by gently pinching between fingers or through trans-illumination of the sac. The sac is opened and the edges are controlled between clips; for this, we usually use Makindo scissors. Fluid in the sac is aspirated as required. If the hernia sac contents have been reduced into the peritoneal cavity spontaneously, there is no need to enlarge the hernia defect, as the hernioscopy will allow visualization of the peritoneal cavity. A purse string suture with 2-0 Vicryl is placed around the open edges of the hernia sac. A blunt trocar is passed through a hernia defect and the Vicryl purse string is tied around this, making an airtight seal. If there is difficulty securing this and the trocar is too mobile, then utilizing a trocar that has pre-placed *ears* where sutures can be secured can help stop it from slipping. Insufflation of the abdominal cavity to 12-15 mmHg using CO_2_ with a flow of 5 L per minute is our standard (insufflation of 10-12 mmHg can be done in patients who are under a spinal anesthetic, as per our team’s experience (Figure 1). The lowest pressure required to visualize the contents properly should be used, and we often use only 8 mmHg in the elderly. A 10 mm zero-degree laparoscope is introduced via the trocar, as shown in Figure 2. This can be changed to 30 degrees if there is difficulty visualizing the abdominal cavity. Diagnostic laparoscopy is performed to ascertain the viability of the hernia contents. If in doubt, further trocars can be introduced under vision to manipulate the abdominal contents and walk the bowel. A 5 mm camera can also be used transabdominally to achieve a different angle of view. Usually, only 5 mm ports are required. If there are concerns regarding a loop of the small bowel, this can be exteriorized through a relatively small incision. After confirmation of viability, or transabdominal management, the hernia defect can be repaired by a traditional low approach either by sutured repair or mesh plug fixation.

## Discussion

Morris et al (2008) [6] reported that spontaneous reduction of hernia contents occurs in around 1% of patients during anesthetic induction. Hernioscopy can be a powerful tool to adequately assess the hernia contents and confirm their viability after this occurs. There are limited reports on hernioscopy as an adjunct in these cases. The technique was first described by Binderow et al. [7] in 1992, and other authors have also published case reports.

A multicentre study in the UK on eight cases by Tebala et al. (2019) [8] reported using hernioscopy as an adjunct in four cases without bowel ischemia and four cases with ischemia. There were no major complications and the authors also emphasized the benefits of avoiding the disruption of fascial planes and large skin incisions. A recent article from Gonullu et al. [9] also highlighted the importance of this highly neglected technique and its additional advantage during the COVID-19 pandemic, such as carrying out the technique under spinal anesthesia and avoiding ventilation.

Other techniques have been described besides simple trocar placement. Mazzota (2020) [10] described the *O-ring* retractor system as an alternative to a metallic trocar. This is especially useful in obese patients where it can help reduce the risk of injury to collateral structures, as well as lower the risk of CO_2_ escape when there is a large hernial orifice and it is difficult to get an air seal. It also reduces aerosol-related infections by reducing gas leaks into the room, helping protect staff.

Using a glove to form a single-incision laparoscopic surgery (SILS) Gloveport hernioscopy is another approach described in 2015 [11]. This allows the introduction of instruments and has been described for umbilical hernias. In our experience, it is unlikely to be of use in a femoral hernia given the rigid boundaries and lack of space. Introducing other trocars transabdominally is more likely to help manipulation providing a larger space between hands and increasing triangulation, and there is little morbidity associated with 5 mm ports. Hernioscopy does not require muscle relaxation or general anesthetics and is a viable technique when performed under spinal anesthesia [12] when there are concerns regarding the patient’s fitness for general anesthesia. In our experience, there are minimal complications and none beyond the standard risks of laparoscopic surgery.

There will occasionally be patients with significant cardiorespiratory disease, where the creation of pneumoperitoneum is not well tolerated. By using the lowest pressure required and insufflating on low flow (5 L/minute or less), with avoidance of the head down position, this can usually be overcome. It is usually not a long procedure in terms of a long period of pneumoperitoneum, and we have had no complications or patients who could not tolerate it in our unit. If the patient truly cannot tolerate pneumoperitoneum, then conversion to open surgery would be required if there is significant doubt regarding the viability of the hernia contents and they cannot be otherwise examined.

Procedural tips

We recommend this technique in cases of spontaneous reduction of previously incarcerated groin hernias, both femoral and inguinal, after induction of general anesthesia, and where Lockwood/Lotheissen incisions were chosen. If, as the emergency surgeon you are widening the femoral ring, please make sure that direction of your scissors is toward the pubic symphysis to avoid injury to the femoral vein and its branches. Careful diathermy use can be considered here. Commercially available balloon trocars are a good option as they form a good seal around most femoral rings if the sac is flimsy. They also secure the trocar if the defect is larger than the trocar and there is excessive movement. It is very difficult to manage any ischemic contents laparoscopically via the groin, so we do not recommend a SILS approach for femoral hernias. Moreover, 5 mm ports should be placed transabdominally. Steep Trendelenburg position is vital for good access, and consideration for positioning should be given. We use a non-slip mattress and a strap across the chest. Thigh straps can get in the way of the laparoscope.

## Conclusions

Hernioscopy is an easy and reliable technique to explore the abdominal cavity, reducing the need for large abdominal incisions and reducing the associated morbidity. The full validation of the hernioscopy technique requires randomized control trials in the future when hernias reduce spontaneously before the contents have been fully examined. There are no systematic reviews to support its use, but the well-known and evidenced benefit of minimally invasive surgery may negate the need for this. Hernioscopy is a technique that all surgeons who operate on groin hernias in an emergency should be familiar with and may wish to utilize in their practice. This technique may allow the surgeon to perform SILS hernioscopy including retrieval of devitalized bowel and resection/repair via a single incision in the groin region, particularly with inguinal hernias where the defect is often larger and more pliable, allowing insertion of a large port. SILS hernioscopy is unlikely to have great benefit in femoral hernias where the primary role of hernioscopy is in diagnostic assessment and facilitating safe transabdominal laparoscopy.
