# Correction: Hernioscopy: A Description of an Adjunct Technique in Emergency Femoral Hernia Surgery

**DOI:** 10.7759/cureus.c286

**Published:** 2025-09-08

**Authors:** Paul W Shuttleworth, Shariq Sabri, Ehtisham Zeb, Andrei Mihailescu

**Affiliations:** 1 General Surgery, Tameside General Hospital, Manchester, GBR

The Ethics Statement and Conflict of Interest Disclosures section of this article has been corrected to indicate that this study did involve human subjects.

